# The Prognostic Value of miR-125b, miR-200c and miR-205 in Primary Cutaneous Malignant Melanoma Is Independent of BRAF Mutational Status

**DOI:** 10.3390/cancers14061532

**Published:** 2022-03-16

**Authors:** Beatriz Sánchez-Sendra, José F. González-Muñoz, Silvia Pérez-Debén, Carlos Monteagudo

**Affiliations:** 1 Department of Pathology, University of Valencia, 46010 Valencia, Spain; sansenbe@uv.es; 2 Biomedical Research Institute INCLIVA, 46010 Valencia, Spain; jgonzalez@incliva.es (J.F.G.-M.); sperez@incliva.es (S.P.-D.); 3 Department of Pathology, Hospital Clínico Universitario de Valencia, 46010 Valencia, Spain

**Keywords:** malignant melanoma, epigenetics, BRAF mutations, miRNAs, prognosis, survival

## Abstract

**Simple Summary:**

Melanoma accounts for the majority of skin cancer-related deaths. On the one hand, most melanomas contain mutations in the BRAF gene (predominantly V600E), and on the other hand, miRNAs modulate different steps in melanoma development and progression, but there are no reports that study the relation between BRAF mutational status and the expression of miRNAs, which is important for an accurate patient prognosis. The aim of our retrospective study was to know whether BRAF mutations influence the prognostic value of miR-125b, miR-200c and miR-205 intratumoral expression in primary cutaneous melanomas. Globally, our results showed that miR-125b, miR-200c and miR-205 expression predicted the clinical outcome of primary melanomas independently of BRAF status. Thus, our findings support that BRAF mutations alone do not predict the risk of metastasis development or melanoma survival and that miR-125b, miR-200c and miR-205 may be considered as accurate prognostic biomarkers in melanoma regardless of BRAF mutational status.

**Abstract:**

BRAF mutations are present in around 50% of cutaneous malignant melanomas and are related to a poor outcome in advanced-stage melanoma patients. miRNAs are epigenetic regulators that modulate different cellular processes in cancer, including melanoma development and progression. However, there are no studies on the potential associations of the genetic alterations of the BRAF gene with miRNA expression in primary cutaneous melanomas. Here, in order to analyze the influence of BRAF mutations in the ability of selected miRNAs to predict clinical outcome and patient survival at the time of diagnosis, we studied the prognostic value of miR-125b, miR-200c and miR-205 expression depending on the BRAF mutational status in fresh, frozen primary tumor specimens. For this purpose, RNA was extracted for studying both BRAF mutations by Sanger sequencing and miRNA expression. Our results indicate that, although there seems to be a slight preference for their predictive ability in the BRAF mutated group, the expression of these three miRNAs serves effectively to predict the clinical outcome of melanoma patients independently of BRAF mutational status at the time of primary tumor diagnosis.

## 1. Introduction

Malignant melanoma causes around 90% of all skin cancer-related deaths, mainly due to metastatic dissemination, with a tumor-specific 10-year-survival of 75–95%, and represents a growing clinical challenge due to its increasing incidence [1,2].

Although most melanoma patients never develop recurrent disease after excision of the primary tumor, identification of the subset of patients who will develop metastatic disease (15–25%) and die from melanoma is of paramount importance. The current staging system of the American Joint Committee on Cancer (AJCC) determines as melanoma prognostic markers Breslow primary tumor thickness, ulceration, lymph node spread and distant metastases [3]. Despite this, clinical and histopathological features alone are not able to accurately predict clinical behavior in melanoma patients.

Moreover, challenges remain in identifying the genetic drivers of melanomagenesis and melanoma progression. Several genes have been identified as genetically altered drivers of melanoma tumorigenesis, such as NRAS, CDNK2A, MITF, PTEN, KIT, GNAQ, GNA11 or CTNNB1 [4,5], but most (60%) melanomas have BRAF gene mutations [6].

BRAF codifies a serine/threonine protein kinase that regulates the MAPK/ERK signaling pathway, involved in the regulation of cell proliferation, differentiation and cell survival. BRAF mutations are located at exon 15, resulting in the substitution of valine at residue 600 (V600). The most frequent BRAF alteration is V600E, triggering MEK and ERK signaling pathway activation, thus driving cancer progression [7]. Unusual BRAF V600R/K/M/G/D mutations can also be detected [5]. BRAF mutations are unlikely to occur as homozygous in clinical melanoma samples, although it has been proposed that some events could mask the heterozygous mutations, such as the amplification of the mutated allele and the loss-of-heterozygosity (LOH) of one of the two alleles, and make them appear as homozygous [8]. It is still unknown if the mutation in BRAF is implicated in the transformation of benign nevi into malignant melanoma or not [9].The presence of a BRAF mutation predicts a poor outcome in advanced-stage melanoma patients [10]. There are different FDA-approved BRAF inhibitors for patients with BRAF V600 mutations in metastatic melanoma resulting in a significant overall survival benefit, although half of the patients showed resistance [11]. Due to the latter, there are therapies that combine BRAF inhibitors with MEK inhibitors in advanced stages of malignant melanoma [11,12].

Epigenetic regulators such as microRNAs (miRNAs) also play a significant role in melanoma development and progression. miRNAs are small noncoding RNAs that can post-transcriptionally regulate genes and cellular processes, including differentiation, cell proliferation, transformation, apoptosis, migration and invasion of melanoma cells [13,14]. In fact, some miRNAs have been defined as prognostic markers of melanoma [15,16,17]. In this regard, our group has recently demonstrated that the intratumoral downregulation of miR-125, miR-200c and miR-205 in primary melanomas contributes to the development of distant metastasis and, therefore, to a shorter survival [18]. Deregulated miRNAs and their targeted genes are associated with the hallmarks of cancer, including melanoma, acting as tumor suppressors or oncogenes [19,20,21], and they may be potential therapeutic targets in melanomas, including those with BRAF mutations [22], by strengthening sensitivity to the standard therapies and immune checkpoint inhibitors [9].

Although there are studies that relate BRAF mutational status and miRNA expression in cultured cells [23,24], few reports have associated miRNAs and individual BRAF mutations with clinical outcomes or have studied their use as prognostic biomarkers in cutaneous melanoma [12,25,26,27], particularly in the progression of primary tumors. Therefore, our main goal was to study the potential prognostic value of the association between BRAF mutational status and miR-125b, miR-200c and miR-205 intratumoral expression in primary melanomas in order to deepen the knowledge of how BRAF genetics and these miRNAs’ expression interact to better predict the clinical outcomes and survival of melanoma patients. In this way, the understanding of BRAF status with the additional epigenetic studies, such as miRNAs, could predict the evolution of melanoma tumors at the time of diagnosis or during treatment [28,29].

## 2. Materials and Methods

### 2.1. Human Melanoma Tissues

In total, 65 fresh, frozen tumor specimens from patients with primary cutaneous malignant melanomas were immediately gathered after surgery from November 2002 to December 2014 at the Anatomic Pathology Department of the Hospital Clínico Universitario, Valencia (Spain). This patient cohort is the same one that we previously studied [18], now with a much longer clinical follow-up and including the additional BRAF mutational status information.

The 2017 American Joint Committee on Cancer (AJCC) staging system was used as a reference to classify these tumors. Clinical follow-up was conducted while paying special attention to the development of distant metastases and melanoma mortality (mean 93, median 97, range 7–188 months). The histological and clinicopathological features of the primary tumors included in this study are summarized in Table 1.

Primary tumor bio-specimens were manually dissected in order to have up to 90% of tumor content. Each fresh tumor was split into two parts. On the one hand, a tumor section close to the thickest area of the tumor was immediately frozen at −80 °C and then used for the extraction of RNA. On the other hand, the remaining fresh tumor tissue was processed for routine histological diagnosis by formalin fixation and paraffin embedding (FFPE). All medical records were revised and clinical follow-up was closed in December 2019. Written informed consent was provided by all patients included in this study.

This study was conducted according to the guidelines of the Declaration of Helsinki, and supervised and approved by the Ethical and Scientific Committees of the Hospital Clínico Universitario of Valencia (Prometeo II/2015/009), and all research was performed in accordance with relevant guidelines and regulations.

### 2.2. RNA Extraction

Total RNA extraction from patient primary melanoma tissues was performed with the mirVana miRNA Isolation Kit (Ambion), according to the manufacturer’s recommendations.

First, melanoma tissues were mechanically disaggregated into tiny portions using a pre-chilled scalpel and then homogenized in 600 µL of Lysis/Binding buffer. The manufacturer’s extraction protocol was followed, with minor modifications. Total RNA was eluted in 60 µL DNase and RNase free dH_2_O. RNA quality and quantity were determined using a NanoDrop One Spectrophotometer (Thermo Fisher Scientific) and stored at −80 °C.

### 2.3. RT-PCR and Sanger Sequencing

Total RNA was reverse-transcribed into cDNA using the MultiScribe Reverse Transcriptase (Thermo Fisher Scientific, Waltham, MA, USA).

Sanger sequencing was performed for BRAF exon 15. Firstly, target gene amplification was performed using forward and reverse specific primers for exon 15 of BRAF gene cDNA and the AmpliTaq Gold^TM^ 360 Master Mix (Thermo Fisher Scientific). Secondly, PCR products were incubated for 15 min at 37 °C, followed by 15 min at 80 °C in the presence of ExoSAP-IT reagent (Thermo Fisher Scientific) for purification. These purified PCR products were the input for the sequencing reactions using the BigDye^®^ Terminator v1.1 Cycle Sequencing Kit (Life Technologies, Carlsbad, CA, USA) according to the manufacturer’s recommendations. Sequencing analyses were carried out using the ABI PRISM 310 Genetic Analyzer (Applied Biosystems, Waltham, MA, USA).

### 2.4. miRNA Quantification

From human primary melanoma samples, relative quantification of mature microRNAs was carried out by reverse transcription quantitative real-time PCR (RT-qPCR), as previously described, from the same RNA samples used for sequencing [18]. Quantitative PCR reactions were set up in triplicate for each sample using a 7900 HT Fast Real- Time PCR system (Lifetechnologies). miRNAs’ Ct values were normalized against those of the small nuclear RNU48, which was used as the endogenous reference control. Samples with an RNU48 mean Ct value outside of the established working range (17 to 23) were not included in the analysis. RT and qPCR negative controls were also included for each tested assay. qPCR results were analyzed with the Expression Suite software (Lifetechnologies) using the ∆Ct method for the relative quantification of miRNA expression.

### 2.5. Statistical Analysis

Data plotting and statistical analysis were conducted using GraphPad Prism V.6.01 (GraphPad Software, Inc., San Diego, CA, USA) and R statistical package. The association between miRNA expression and BRAF genetic status (WT or mutated), metastasis development and exitus was analyzed using two-tailed Mann–Whitney tests. Non-parametric survival analysis was performed to assess distant metastasis-free survival (DMFS) and melanoma-specific survival (MSS) using the Kaplan–Meier method. Log-rank test was used to statistically compare the curves. DMFS and MSS were defined as the length of the period from the initial melanoma diagnosis date to the date of distant metastasis development and last follow-up (censored) or death from melanoma (event), respectively. Logistic regressions were constructed depending on BRAF mutational status in order to model the probability of distant metastasis and exitus. Univariate Cox proportional hazard models were constructed for both BRAF WT and BRAF mutated primary tumor subgroups considering each miRNA’s expression (miR-125b, miR-200c or miR-205) as a prognostic variable to be analyzed independently. For all statistical tests, a *p*-value of less than 0.05 was considered statistically significant.

## 3. Results

### 3.1. BRAF Mutational Status in Primary Melanomas

**Figure 1:**
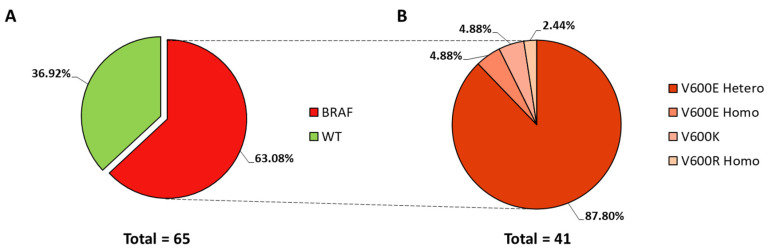
Pie charts representing the percentage of wild-type (WT) and BRAF mutated samples. (**A**) Percentage of BRAF mutated samples including all variants. (**B**) Percentage of BRAF mutation variants detected in exon 15 (V600) regarding total BRAF mutated samples. Homo, homozygotic; hetero, heterozygotic.

Mutations in BRAF exon 15 (V600) were detected in 41 (63.08%) of 65 primary melanoma tissues (Figure 1A). Among these, BRAF V600E was found as the most frequent variation (considering heterozygotic and homozygotic modifications), present in 38 samples of 41 (92.68%). In addition, there were two samples with V600K mutations (4.88%) and one sample with the mutation V600R (2.44%) (Figure 1B).

### 3.2. miRNA Expression Profile in BRAF Mutated and BRAF Wild-Type Primary Melanomas

**Figure 2:**
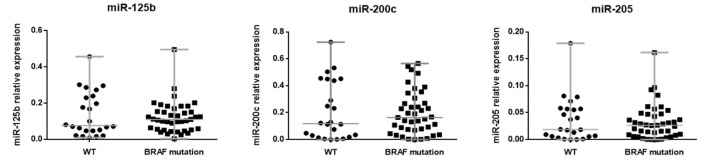
Relative expression of miR-125b, miR-200c and miR-205 according to the mutational status of BRAF (WT or any BRAF mutation).

Studying the BRAF mutational status of the primary tumors, no significant differences in miR-125b, miR-200c and miR-205 expression depending on the mutational status of BRAF were found. Median expression values of miR-125b, miR-200c and miR-205 were slightly lower in WT samples in comparison with those showing any BRAF mutation, but without reaching a significant difference (*p*-values 0.9516, 0.7207 and 0.9409, respectively) (Figure 2).

### 3.3. miRNA Expression Profile in BRAF Wild-Type Primary Metastatic Melanoma

Because no significant differences were observed in the expression profiles of miR-125b, miR-200c and miR-205 between BRAF wild-type and BRAF mutated primary melanomas, we analyzed whether the expression profiles of these three miRNAs were independently able to discriminate among primary tumors having metastatic potential and those without this ability in the BRAF WT and BRAF mutated groups.

**Figure 3:**
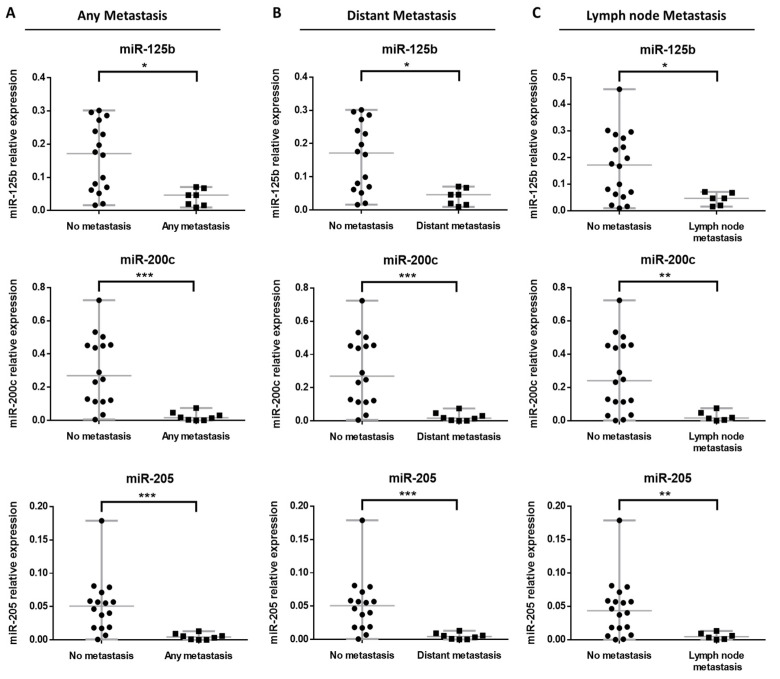
Relative expression of miR-125b, miR-200c and miR-205 according to the metastatic behavior of BRAF WT primary tumors for (**A**) any metastasis, (**B**) distant metastasis and (**C**) lymph node metastasis. Significant at * *p*-value < 0.05, ** *p*-value < 0.01 and *** *p*-value < 0.001.

Considering the development of any metastasis (lymph node or distant metastasis), BRAF WT primary tumors that metastasized showed significantly lower expression of miR-125b, miR-200c and miR-205 than BRAF WT tumors that did not metastasize (*p*-value 0.0382, 0.0001 and 0.0002, respectively) (Figure 3A). Patients with BRAF WT tumors that metastasized had at least one metastasis to a distant organ, so the expression profile for distant metastasis was identical (Figure 3A,B).

In addition, primary BRAF WT tumors with lymph node metastasis also showed significantly lower expression of miR-125b, miR-200c and miR-205 in comparison with WT tumors for BRAF that did not metastasize (*p*-value 0.0224, 0.0044 and 0.0074, respectively) (Figure 3C).

### 3.4. miRNA Expression Profile in BRAF Mutated Primary Metastatic Melanoma

**Figure 4:**
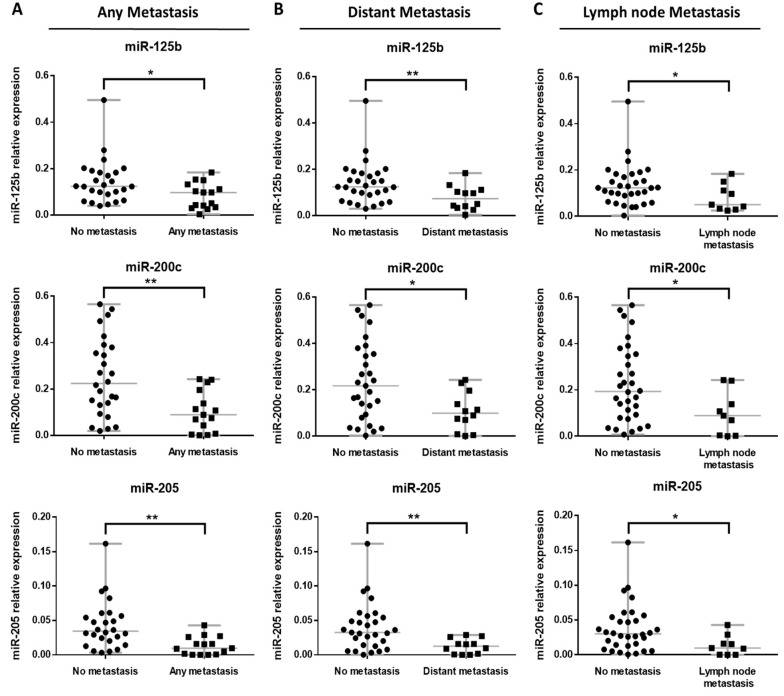
Relative expression of miR-125b, miR-200c and miR-205 according to the metastatic behavior of BRAF mutated primary tumors for (**A**) any metastasis, (**B**) distant metastasis and (**C**) lymph node metastasis. Significant at * *p*-value < 0.05, ** *p*-value < 0.01.

When studying any BRAF mutation and considering any metastasis, primary tumors that metastasized presented significantly lower expression of miR-125b, miR-200c and miR-205 than BRAF mutated tumors that did not metastasize (*p*-value 0.0166, 0.0035 and 0.0004, respectively) (Figure 4A). BRAF mutated tumors showed an important decrease in miR-125b, miR-200c and miR-205 levels (*p*-value 0.0098, 0.018 and 0.0022, respectively) when distant metastasis occurred in comparison with miRNA levels of tumors that did not (distant) metastasize (Figure 4B). Primary tumors with any BRAF mutation developing lymph node metastasis had also significantly lower miR-125b, miR-200c and miR-205 (*p*-value 0.0499, 0.0268 and 0.017, respectively) compared to tumors not presenting lymph node metastasis (Figure 4C).

No significant differences were found in the expression profiles of miR-125b, miR-200c and miR-205 between BRAF mutated tumors that developed in transit metastasis and those that did not develop it (*p*-value 0.4863, 0.3057 and 0.1867, respectively, data not shown).

### 3.5. Melanoma-Specific and Distant Metastasis-Free Survival for Primary Melanomas

**Figure 5:**
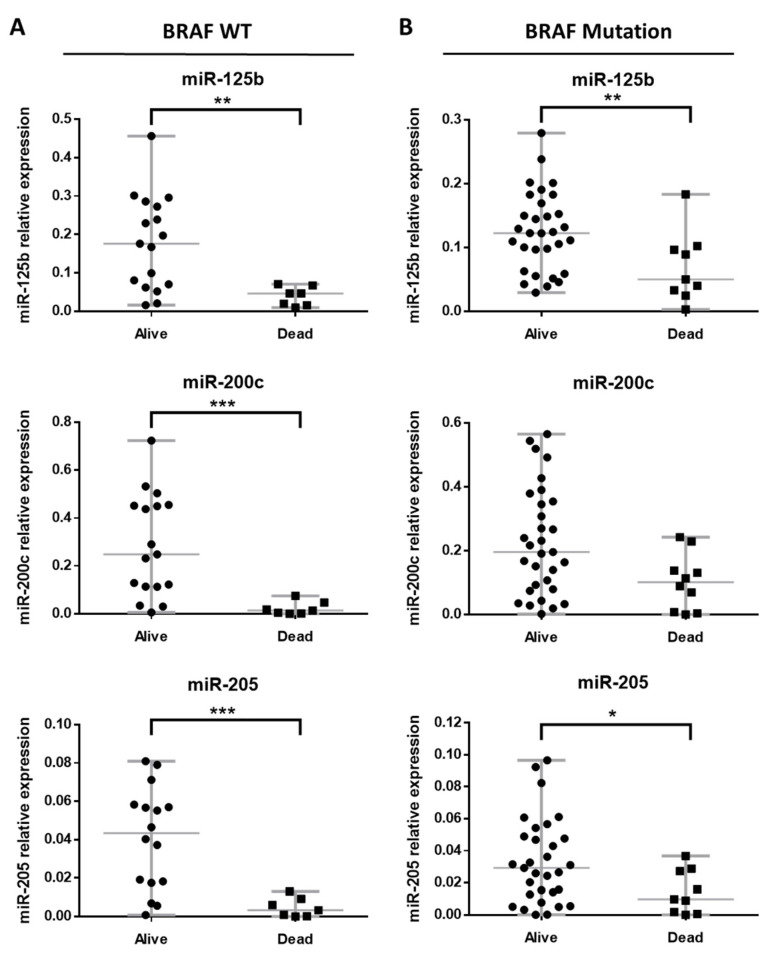
Association of relative expression of miR-125b, miR-200c and miR-205 in primary melanomas according to melanoma-specific death for (**A**) BRAF WT tumors and (**B**) any BRAF mutated tumors. Significant at * *p*-value < 0.05, ** *p*-value < 0.01 and *** *p*-value < 0.001.

Among the BRAF WT primary melanomas, 33.3% (8/24) further progressed to distant metastatic tumors, and 87.5% (7/8) of them died of melanoma. Of the BRAF mutated primary melanomas, 51.22% (21/41) developed distant metastasis and 75% (9/12) of them finally died of melanoma. There seems to be an association between miR-125b, miR-200c and miR-205 expression levels and death of melanoma (exitus) occurrence. Expression levels of miR-125b, miR-200c and miR-205 were significantly lower in BRAF WT primary tumors of patients who died from melanoma (*p*-value 0.0025, 0.0004 and 0.0008, respectively) (Figure 5A). This tendency was also observed in BRAF mutated primary tumors of patients who died from melanoma, but the decrease was significant only for miR-125b and miR-205 (*p*-value 0.0078 and 0.0398, respectively) (Figure 5B).

**Figure 6:**
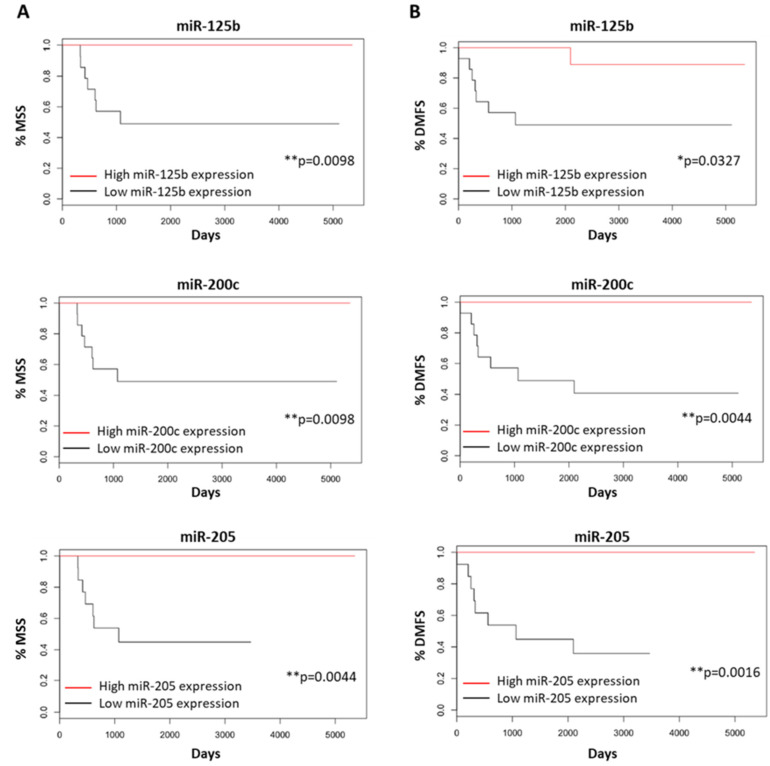
Kaplan–Meier curves for MSS and DMFS of BRAF WT primary melanoma patients depending on miR-125b, miR-200c and miR-205 expression being below (black) or over (red) their corresponding median expression values. Influence of miR-125b, miR-200c and miR-205 expression affecting (**A**) MSS and (**B**) DMFS of patients, respectively. Significant at * *p*-value < 0.05, ** *p*-value < 0.01.

In the survival analysis, the BRAF WT primary melanoma population was dichotomized into patients with high or low miR-125b, miR-200c or miR-205 expression depending on their corresponding median values. In the Kaplan–Meier survival analysis, both melanoma-specific survival (MSS) and distant metastasis-free survival (DMFS) were significantly longer in patients with BRAF WT primary melanomas showing miR-125b (MSS rate = 100% vs. 48.98%, *p*-value = 0.0098 and DMFS rate = 88.89% vs. 48.98%, *p*-value = 0.0327), miR-200c (MSS rate = 100% vs. 48.98%, *p*-value = 0.0098 and DMFS rate = 100% vs. 40.82%, *p*-value = 0.0044) and miR-205 (MSS rate = 100% vs. 44.81%, *p*-value 0.0044 and DMFS rate = 100% vs. 35.89%, *p*-value = 0.0016) expression levels above the median. Survival rates were close to 100% in most cases with high miR-125b, miR-200c and miR-205 expression (Figure 6).

**Figure 7:**
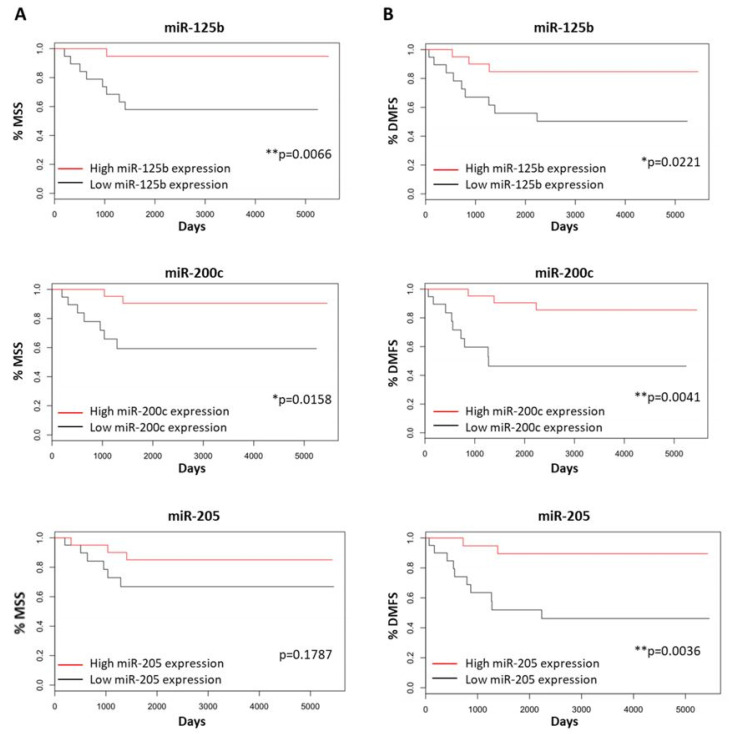
Kaplan–Meier curves for MSS and DMFS of BRAF mutated primary melanoma patients depending on miR-125b, miR-200c and miR-205 expression being below (black) or over (red) their corresponding median expression values. Influence of miR-125b, miR-200c and miR-205 expression affecting (**A**) MSS and (**B**) DMFS of patients, respectively. Significant at * *p*-value < 0.05, ** *p*-value < 0.01.

In patients with BRAF mutated primary melanomas, MSS and DMFS were significantly longer in patients showing miR-125b (MSS rate = 94.74% vs. 57.89% *p*-value = 0.0066 and DMFS rate = 84.71% vs. 50.33%, *p*-value = 0.0221) and miR-200c (MSS rate = 90.48% vs. 59.32%, *p*-value = 0.0158 and DMFS rate = 85.45% vs. 46.39%, *p*-value = 0.0041) expression levels above the median. Patients with any BRAF mutation showing miR-205 expression levels over the median also revealed a DMFS that was significantly longer (DMFS rate = 89.47% vs. 46.20%, *p*-value = 0.0036) but not a significantly longer MSS (Figure 7).

### 3.6. miRNAs as Potential Independent Biomarkers for Clinical Outcomes

Logistic regression analysis was performed to determine if the mutational status of the BRAF gene could be a predictive factor for the development of distant metastasis and melanoma mortality, considering all primary melanoma samples. This analysis showed that BRAF status alone is not able to predict the probability of further developing either distant metastasis or exitus (Table 2).

Cox analysis was performed in BRAF WT and BRAF mutated primary melanomas to elucidate if miR-125b, miR-200c and miR-205 had, on their own, a relevant influence on distant metastasis-free survival and melanoma-specific survival as independent prognostic factors of clinical outcomes (DMFS and MSS). In BRAF WT melanomas, miR-205 was found to significantly explain only distant metastasis-free survival (*p*-value = 0.046). In BRAF mutated ones, miR-125b and miR-205 were significant to predict both DMFS (*p*-value = 0.017 and 0.008, respectively) and MSS (*p*-value = 0.025 and 0.047, respectively); meanwhile, miR-200c only explained DMFS (*p*-value = 0.016). As the miRNA hazard ratios indicate, the lower the miRNA expression level, the higher the risk of distant metastasis development over time and, therefore, the shorter the patient survival (Table 3).

As primary tumor Breslow thickness and ulceration continue to represent important prognostic factors that define T1 primary cutaneous melanomas and that are incorporated into the melanoma patient staging, we analyzed the prognostic value of patient staging in relation to the risk of developing distant metastasis and death from melanoma in both BRAF WT and BRAF mutated groups. For this analysis, patient staging was grouped into three categories (stages 0 and I; stage II; stages III and IV) and data were analyzed using the Pearson chi square test. These results showed that the prognostic value of patient staging remained significant for distant metastasis in both BRAF WT (*p* 0.00012) and BRAF mutated primary tumors (*p* 0.00604) and also for exitus in the BRAF WT (*p* 0.00039) versus BRAF mutated group (*p* 0.04574) and that there were no differences in the prognostic value of miRNAs in relation to stage and BRAF mutational status.

## 4. Discussion

BRAF is one of the most commonly mutated genes in melanoma, the percentage of mutations ranging from 40 to 60%. Moreover, 80% of these BRAF mutations are in the position V600E [30]. These data support the results obtained in our study, where 63.08% of melanoma samples had a BRAF mutation, the V600E alteration being the most common (92.68%) [11].

There is a discrepancy regarding the prognostic value of mutations in the BRAF gene in melanoma patients. Although some studies have found that BRAF mutations are an independent favorable prognostic factor, showing better overall survival than BRAF wild-type tumors [9,31,32], other investigators suggest either that the presence of any mutation in the BRAF gene increases the risk of death in melanoma patients [31,33] or that there is no correlation between BRAF mutation and prognosis [34,35].

Logistic regression depending on BRAF status was not significant for distant metastasis and death from melanoma, denoting that, in our study, BRAF status alone did not predict the risk of adverse events. Therefore, as Mar et al. suggest [33], additional prognostic variables are necessary to predict the clinical outcome of primary melanomas, independently of BRAF status. Khaliq et al. defend that epigenetic mechanisms could influence the progression of BRAF mutated cancers and MAPK inhibitor resistance, so knowledge of the epigenetic changes might enable us to know the dependence on BRAF status and find new effective therapies [29]. For this reason, selected and previously described miRNAs such as miR-125b, miR-200c and miR-205 could serve as prognostic biomarkers in melanoma [18].

Regarding miRNA expression analysis, initially, we found that the expression of miR-125b, miR-200c and miR-205 was not dependent on the mutational status of BRAF in primary melanoma tumors because there were no significant differences in miRNA levels between wild-type and mutated BRAF, although Pinto et al. described a general downregulation of 14 miRNAs in BRAF mutated tumors in comparison with BRAF WT [27]. Therefore, we decided to study the expression of these three miRNAs and their relation with clinical outcomes (distant metastasis development and death of melanoma) separately in BRAF WT and BRAF mutated primary tumors, in order to detect differences in the miRNAs’ behavior between both groups and to see if miRNAs were able to discriminate properly among primary tumors that will develop metastasis and, among the latter, those that will die of melanoma, in both groups.

Previous works had associated lower levels of miR-200c and miR-205 in primary melanomas with shorter survival and proposed them as prognostic biomarkers for high-risk melanoma patients [36,37]. With regard to miR-125b, it has been described as a dual microRNA, which can act as a tumor promoter and suppressor [38]. Downregulated miR-125b in FFPE and fresh frozen tissues has been associated with lower survival and poor prognosis in malignant melanoma [18,39]. However, it has to be noted that increased miR-125b expression has been found in melanoma cells resistant to BRAF inhibitors and in tumor samples from BRAF-inhibitor-treated patients [40,41]. Upregulation of miR-125b was also found in patients treated with an mTOR inhibitor and VEGF blockade with Temsirolimus and Bevacizumab, respectively [42,43].

According to our results, there seems to be a significant association between lower expression levels of miR-125b, miR-200c and miR-205 and metastasis development and even death from melanoma occurrence within both study groups. In detail, in our study, the downregulation of miR-125b, miR-200c and miR-205 was observed in primary melanomas that further metastasized (distant and lymph node metastasis) and in primary tumors of patients who died of melanoma, for both the BRAF WT and BRAF mutated subsets. The only case in which such a downregulation was found not significant was for miR-200c expression in BRAF mutated primary tumors in relation to melanoma-specific death. These results were consistent with those obtained in the Kaplan–Meier survival analysis, where survival rates (DMFS and MSS) were significantly lower in primary tumors of patients showing lower expression of these three miRNAs, except for miR-205 in BRAF mutated cases in relation to MSS. A correlation of low levels of these miRNAs with shorter melanoma-specific and distant metastasis-free survival (MSS and DMFS) was previously described in fresh frozen primary melanomas without taking into consideration BRAF status [18]. Consequently, these new findings indicate that the influence of these three miRNAs in the progression of melanoma is independent of BRAF mutational status. We also analyzed the prognostic value of patient staging with the risk of developing distant metastasis and death from melanoma. We observed that patient staging had prognostic value by itself in both BRAF W and BRAF mutated primary tumors, and that there were no significant differences in the prognostic value of miRNAs in relation to tumor staging.

Although lower expression of these three miRNAs discriminated well distant melanoma development, death of melanoma and lower survival rates, similarly in both groups, independently of BRAF mutational status, not all of them resulted to be significant prognostic factors for MSS and DMFS in the respective groups. Cox regressions showed that in BRAF native patients, only miR-205 was an independent prognostic factor and influenced distant metastasis-free survival. However, in patients with melanomas harboring BRAF gene mutations, the risk of developing a distant metastasis over time was significantly affected by miR-125b, miR-200c and miR-205 levels independently. Moreover, miR-125b and miR-205 expression were also independent prognostic factors for melanoma-specific survival (MSS) in the BRAF mutated group. Therefore, the expression of these three miRNAs seems to fit better as an independent prognosis factor of DMFS and MSS in BRAF mutated than in BRAF WT tumors. BRAF mutated primary tumors showing low expression of these three miRNAs have a higher risk of earlier developing metastasis or dying from melanoma with regard to BRAF WT tumors. However, the HR values for the studied miRNAs were near to 1, pointing out that the risk added for each miRNA is very low, less than 6% (e.g., HR value of 0.944 for miR-205 in BRAF mutated group in DMFS analysis).

Therefore, we observed that miR-205 may serve as a potential biomarker for the risk of developing distant metastasis over time, which is in line with previous reports [18,26], and now, according to our current work, it stands up as a prognostic biomarker that influences and predicts clinical outcomes regardless of BRAF mutational status. Furthermore, miR-125b may be used as a specific biomarker for BRAF mutated primary melanomas to predict distant metastasis-free survival and melanoma-specific survival, which suggests that it could be involved in melanoma progression [44]. In addition, the loss of miR-200c, a tumor suppressor in melanoma, seems to shorten the distant metastasis-free survival only in BRAF V600 mutated tumors [45].

Hence, our findings support that BRAF mutational status alone does not have prognostic value in primary melanomas in order to predict the risk of developing metastasis or death and, therefore, it is necessary to include other prognostic variables such as the expression of miR-125b, miR-200c and miR-205. Although these miRNA levels can differentiate well at the time of primary tumor diagnosis between patients with and without poor clinical outcomes independently of BRAF status, there seems to be a slight preference for their predictive ability in the BRAF mutated group. However, the risk of metastasis development and death from melanoma over time added independently by each miRNA is considerably low, so further research taking into consideration the influence of the expression of these miRNAs together with other potential prognostic variables, either genetic or clinicopathologic, in a multivariant model will be convenient to improve clinical outcome predictions. In this way, miR-125b, miR-200c and miR-205 may be considered as prognostic biomarkers in primary melanomas to predict clinical outcomes and survival accurately, but other prognostic factors could also influence an increased risk of metastasis or death from melanoma. Further studies should be performed to clarify the influence of miR-125b, miR-200c and miR-205 in the treatment of melanoma patients with BRAF inhibitors.

## 5. Conclusions

The results of this study suggest that BRAF mutational status alone does not predict the clinical outcomes in primary cutaneous melanoma and that the influence of miR-125b, miR-200c and miR-205 in the progression of melanoma is independent of BRAF mutations. Therefore, miR-125b, miR-200c and miR-205 could be used as prognostic biomarkers for metastasis development and melanoma survival in a BRAF mutational status-independent manner. Further investigation would be needed to determine the role of these miRNAs, miR-125b, miR-200c and miR-205, in melanoma patients treated with BRAF inhibitor therapy.

## Figures and Tables

**Table 1:** Primary melanomas’ histological and clinical features.

Primary Melanomas (Total Number: 65)		
Variable	Number of Patients	(%)
Breslow thickness (mm)		
≤1	26	40.0
1.01–2.00	14	21.5
2.01–4.00	8	12.3
>4	17	26.2
Ulceration		
Absent	48	73.8
Present	17	26.2
Mitosis/mm^2^		
0	19	29.2
≥1	46	70.8
Growth phase		
Radial	20	30.8
Vertical	45	69.2
Location		
Head and neck	7	10.8
Limbs	23	35.4
Trunk	34	52.3
Gender		
Female	42	64.6
Male	23	35.4
Histological type *		
ALM	5	7.7
LMM	5	7.7
NM	9	13.8
SSM	46	70.8
Age at diagnosis (years)		
≤65	34	52.3
>65	31	47.7
In-transit metastasis		
Absent	55	84.6
Present	10	15.4
Lymph node metastasis		
Absent	50	76.9
Present	15	23.1
Distant metastasis		
Absent	45	69.2
Present	20	30.8
Any metastasis		
Absent	42	64.6
Present	23	35.4
Melanoma-specific survival		
Alive	49	75.4
Dead	16	24.6
Follow-up (months)		
Mean, Range	93	(7–188)

* ALM (Acral Lentiginous Melanoma), LMM (Lentigo Maligna Melanoma), NM (Nodular Melanoma) and SSM (Superficial Spreading Melanoma).

**Table 2:** Univariate logistic regression analysis for distant metastasis and exitus depending on BRAF mutational status.

Variables	Estimate	Std. Error	z Value	Pr (>|z|)
Distant Metastasis				
Intercept	−0.754	0.429	−1.76	0.079
BRAF mutational status	−0.195	0.547	−0.36	0.721
Exitus				
Intercept	−0.945	0.445	−2.12	0.034 *
BRAF mutational status	−3.385	0.582	−0.66	0.509

* *p*-value < 0.05.

**Table 3:** Univariate Cox regression analysis of miRNAs for DMFS and MSS.

miRNAs	HR (exp(b))	95% CI	*p*-Value
BRAF WT			
Distant metastasis-free survival (DMFS)			
miR-125b	0.995	0.987–1.002	0.148
miR-200c	0.980	0.961–1	0.052
miR-205	0.904	0.818–0.999	0.046 *
Melanoma-specific survival (MSS)			
miR-125b	0.982	0.963–1	0.055
miR-200c	0.981	0.961–1.002	0.070
miR-205	0.909	0.823–1.005	0.063
BRAF Mutated			
Distant metastasis-free survival (DMFS)			
miR-125b	0.986	0.975–0.998	0.017 *
miR-200c	0.994	0.988–0.999	0.016 *
miR-205	0.944	0.905–0.985	0.008 **
Melanoma-specific survival (MSS)			
miR-125b	0.984	0.970–0.998	0.025 *
miR-200c	0.995	0.989–1	0.066
miR-205	0.957	0.916–0.999	0.047 *

* *p*-value < 0.05, ** *p*-value < 0.01.

## Data Availability

The data presented in this study are available from the corresponding author upon reasonable request.
